# Course of respiratory allergy by treatment strategy based on German routine data

**DOI:** 10.1007/s40629-017-0027-x

**Published:** 2017-05-30

**Authors:** Ann-Kathrin Weschenfelder, Ludger Klimek, Norbert Mülleneisen, Harald Renz, Wolfgang Wehrmann, Thomas Werfel, Eckard Hamelmann, Jürgen Wasem, Janine Biermann

**Affiliations:** 1 Institute for Health Care Management and Research, University of Duisburg-Essen, Essen, Germany; 2 Thea-Leymann-Str. 9, 45127 Essen, Germany; 3 Centre for Rhinology and Allergology, Wiesbaden, Germany; 4 Asthma and Allergy Centre, Leverkusen, Germany; 5 Institute of Laboratory Medicine and Pathobiochemistry, Molecular Diagnostics, Philipps University Marburg, Marburg, Germany; 6 Dermatological Clinic Prof. Wehrmann, Dr. Rödder-Wehrmann and Colleagues, Münster, Germany; 7 Division of Immunodermatology and Allergy Research, Department of Dermatology and Allergy, Hannover Medical School, Hannover, Germany; 8 Children’s Center Bethel, Protestant Hospital Bielefeld and Allergy Center, Ruhr-University, Bochum, Germany

**Keywords:** Disease progression, Drug therapy, Desensitization, immunologic, Sublingual immunotherapy, Rhinitis, allergic, Asthma

## Abstract

**Purpose:**

Allergic respiratory diseases represent a global health problem. The two major treatment strategies are symptom treatment and specific immunotherapy (SIT). SIT is considered the only causal treatment option available with the ability to alter the course of the disease. This study aims to describe the course of disease and medication of respiratory allergy across treatment strategies and disease groups.

**Methods:**

The analysis is based on routine data from a German statutory health insurance. The patient cohort is observed from 2007–2012. For each year based on assured outpatient diagnoses patients are assigned to a disease group: rhinitis, asthma or both diseases. Additionally, prescribed medication is considered. Treatment comparisons are based on matched pairs.

**Results:**

The study population comprises 165,446 patients with respiratory allergy. In 2007 the most frequent disease group is rhinitis (70%), followed by asthma (16%) and both diseases (14%). During the observation period a second allergic respiratory diagnosis occurs only in about 12% of rhinitis patients and 28% of asthma patients. In about 50% of patients with both diseases one of the diagnoses is omitted. These patients are more likely to no longer report their asthma diagnosis when receiving immunotherapy compared to symptom treatment. Furthermore immunotherapy reduces the frequency of asthma medication use.

**Conclusions:**

Results of detailed analysis of diagnoses reflect the alternating nature of allergic diseases. Although limited by accuracy of documentation and the lack of clinical information, the comparison of treatment strategies shows some advantages of immunotherapy regarding course of disease and asthma medication use.

## Introduction

Occurring in both developed and developing countries and across all ethnic groups and ages allergic respiratory diseases represent a global health problem [1]. Their global prevalence ranges between 5% and 40% [2, 3]; in Germany the prevalence for asthma is 9% and for allergic rhinitis it is 15% [4]. Respiratory allergy patients experience symptoms and impairments in daily life activities, social life, sleep and ability to work reducing their quality of life [5–7].

Specific immunotherapy (SIT) is able to influence the underlying immunological mechanisms of allergy [8]. It can be applied as subcutaneous injection (SCIT) or sublingual treatment (SLIT) [9]. Additionally, various pharmaceuticals are available to treat symptoms of allergic respiratory diseases [1, 10]. While allergen avoidance can be seen as further treatment strategy it is often not completely feasible in daily life. Therefore, specific immunotherapy is considered the only treatment for respiratory allergy with the ability to alter the natural course of the disease [8].

This study aims to first describe the course of allergic respiratory diseases under different treatment strategies and, second to compare the course of disease and the use of prescription drugs between the treatment strategies.

## Materials and methods

This study is based on routine data of a nationwide German statutory health insurance (SHI) company available for the years 2005–2012. The study population is derived from all insured persons in 2007. This cohort is then observed from the baseline year 2007 until 2012, while 2005 and 2006 data are used for population selection.

Diagnoses are available as International Classification of Diseases (ICD) codes version 10 German modification (GM). Patients are considered to have allergic respiratory disease when they received at least one assured outpatient diagnosis of J30.1 “allergic rhinitis due to pollen”, J30.2 “other seasonal allergic rhinitis”, J30.3 “other allergic rhinitis” or J30.4 “allergic rhinitis, unspecified” for allergic rhinitis or J45.0 “predominantly allergic asthma” or J45.8 “mixed asthma” for allergic asthma within the year under consideration [11, 12]. The choice of these asthma ICD codes ensures an allergic component of the disease.

Based on their diagnoses insured persons are grouped to the following disease groups for each year: isolated allergic rhinitis (AR), isolated allergic asthma (AA) and allergic rhinitis with concomitant allergic asthma (ARAA). This third group of patients having both diseases is established to account for differences in patients’ quality of life and treatment requirements. These differences result from the negative influence of having both diseases on the severity of each single disease [13, 14]. The course of disease is analyzed based on these three disease groups.

Disease-related prescription drugs are identified using the Anatomical Therapeutic Chemical (ATC) codes and their subordinated national drug codes. Relevant active ingredients are listed in Table 1. The analysis excludes over-the-counter drugs as these are only documented for insured persons under the age of 12. Based on their prescriptions, insured persons are assigned to the following medication groups for each year: no medication, asthma medication, rhinitis medication and medication for both diseases.

**Table 1:** Active ingredients for symptom treatment of allergic respiratory diseases relevant to the study

ATC code	Active pharmaceutical ingredient group
*Rhinitis*	
R01AD	Topical corticosteroids
*Asthma*	
R03AA	Alpha- and beta-adrenoreceptor agonists^a^
R03AC	Selective beta-2-adrenoreceptor agonists^a^
R03AK	Adrenergics in combination with corticosteroids or other drugs, excluding anticholinergics^a^
R03BA	Glucocorticoids^a^
R03BB	Anticholinergics^a^
R03BC	Antiallergic agents, excl. corticosteroids^a^
R03DC03	Montelukast
R03DX05	Omalizumab

*ATC* anatomical therapeutic chemical classification

^a^Inhalants

Treatment strategies under investigation are specific immunotherapy (SIT) with symptom treatment as needed and symptom treatment alone (ST). Within specific immunotherapy subcutaneous and sublingual application are distinguished. Immunotherapy prescriptions are identified through ATC codes and national drug codes for inhalant allergen extracts.

The study cohort is established based on 2007 data. Inclusion criteria are having an allergic respiratory disease diagnosis and data being available in 2005–2012. Exclusion criteria are age younger than five years and SIT prescription in 2005 or 2006. Insured persons under the age of five are excluded because medical guidelines do not recommend the use of SIT below this age [15]. To avoid possible bias from recently conducted courses of SIT only insured persons starting immunotherapy are analyzed by excluding those with immunotherapy prescription in 2005 or 2006.

The selected population is split into SIT and ST treatment strategy based on their prescriptions in 2007. In the ST group a further exclusion criterion is having a SIT prescription during the observation period (2008–2012). By splitting the SIT group into SCIT and SLIT insured persons with prescriptions for both application forms are excluded. To achieve homogeneity of analyzed treatment phases within each type of SIT, only the most frequently recorded treatment duration is taken into account. Selection is based on reported treatment duration due to the lack of a definite recommendation in German medical guidelines [15]. Therefore insured persons are excluded if their treatment duration is shorter than the minimum requirement of three years and not equal to the most frequently reported duration.

**Fig. 1:**
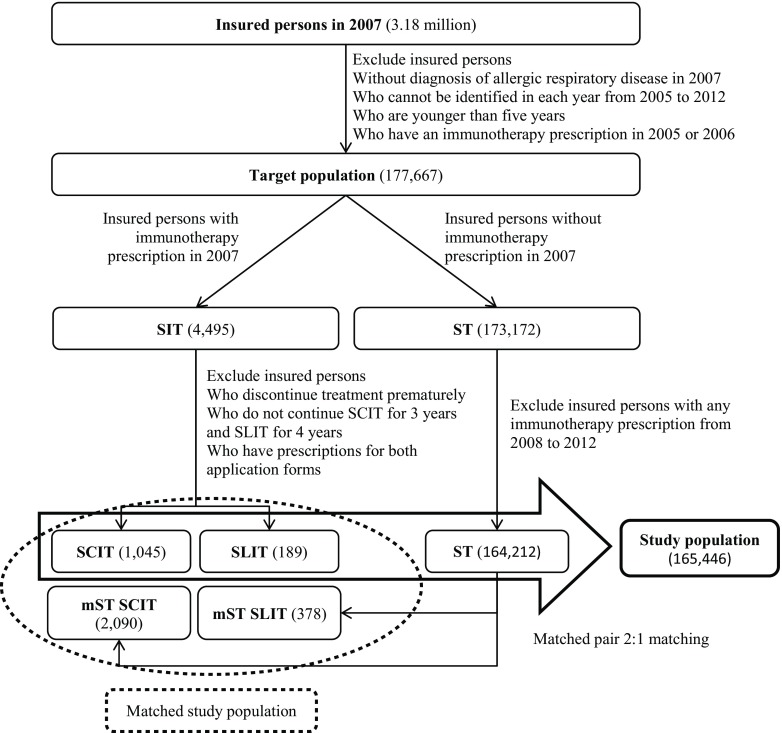
Population selection algorithm (*SCIT* subcutaneous immunotherapy, *SLIT* sublingual immunotherapy, *ST* symptom treatment, *mST* matched symptom treatment group)

To compare the course of disease and medication between the treatment strategies a matched study population is defined. Using an exact matched pair method without replacement each person in the SCIT and SLIT group is assigned to two persons of the symptom treatment group. The matching criteria are the following: five year age groups, gender, and baseline disease and medication group (Fig. 1 summarizes the population selection process).

Statistical analyses are conducted using IBM SPSS version 21.0. All group differences are tested at a significance level of 5% applying the Chi^2^-test except of the number of medication-free years which are assessed by Mann–Whitney U‑test. In case of small numbers of observations leading to invalid Chi^2^-test results the Fishers Exact test is applied. Odds ratios are calculated using univariate logistic regression.

## Results

### Population

In 2007 a total of 177,667 insured persons (mean age 44 ± 21 years, 66% female) met the inclusion criteria. Of those about 2.5% started SIT in 2007 (2% SCIT, 0.5% SLIT and 0.03% both application forms). Treatment discontinuation rate before the required minimum period of 3 years is 41% for SCIT and 52% for SLIT. Most discontinuations occur after the first year of treatment (64% SCIT, 73% SLIT); however, some of those resume therapy at a later time (27% SCIT, 7% SLIT). The most frequently reported treatment duration is 3 years for SCIT (42% of adherent patients) and 4 years for SLIT (43% of adherent patients).

After applying the treatment-group-specific exclusion criteria the study population comprises 165,446 insured persons (Table 2). The largest proportion of this population (70%) is assigned to the disease group isolated rhinitis followed by isolated asthma (16%) and both diseases (14%).

**Table 2:** Study population by disease group and treatment strategy

Disease group	Study population			
	SCIT	SLIT	ST	Total
AR	705	129	115,379	116,213
ARAA	307	51	20,781	21,139
AA	33	9	28,052	28,094
Total	1045	189	164,212	165,446

*AR* allergic rhinitis, *AA* allergic asthma, *ARAA* allergic rhinitis and allergic asthma, *SCIT* subcutaneous immunotherapy, *SLIT* sublingual immunotherapy, *ST* symptom treatment

The pool of eligible matching partners for the SCIT and SLIT groups contains the entire symptom treatment group (“ST” column in Table 2). The matching was successfully conducted resulting in matched symptom treatment (mST) groups twice the size of the SCIT and SLIT group.

### Course of disease across treatment strategies

In patients with isolated rhinitis at baseline the most frequent courses of disease are omission of rhinitis diagnosis (34%), continuous diagnosis of rhinitis (32%) and intermittent rhinitis diagnosis and diagnosis-free years (21%). Less than 20% of patients with rhinitis document asthma as additional diagnosis during the observation period. Of those the largest proportion shows both diseases concomitantly rather than periods of isolated asthma and isolated rhinitis (AR and AA) or periods of all three disease groups (AR, AA and ARAA) interchangeably (Table 3).

**Table 3:** Five year course of disease of insured persons with isolated rhinitis (*AR*) at baseline by treatment strategy

Description	SCIT	mST SCIT	SLIT	mST SLIT	ST total
*N* total	705 (in %)	1410 (in %)	129 (in %)	258 (in %)	115,379 (in %)
*Only AR diagnosis* ^*c*^	*84*	*85*	*82*	*90*	*88*
Continuous AR diagnosis^a,c^	32	27	34	27	32
Intermittent AR diagnosis^a,d^	15	22	13	22	21
Omission of AR diagnosis	36	36	35	41	34
*Additional AA diagnosis* ^*c*^	*16*	*15*	*18*	*10*	*12*
*AR followed by ARAA diagnosis* ^*a*^	*13*	*10*	*14*	*6*	*9*
AR followed by ARAA and omission	3	2	3	2	2
AR followed by ARAA with omission of AA	4	3	5	1	3
Intermittent AR and ARAA	1	1	0	1	1
AR followed by ARAA	5	4	6	3	4
*AR and AA diagnoses* ^*b*^	*2*	*3*	*2*	*2*	*2*
AR followed by AA and omission	1	1	2	1	1
AR followed by AA and AR	0.4	0.4	0	1	0.2
AR followed by AA	1	1	0	0.4	1
*AR, AA and ARAA diagnoses*	*2*	*2*	*2*	*2*	*1*
AR followed by AA and ARAA	0.1	0.4	0	0.4	0.2
AR followed by ARAA and AA	1	1	1	0	1
Intermittent diagnoses of AR, AA and ARAA	1	1	2	1	1

*AR* allergic rhinitis, *ARAA* allergic rhinitis and allergic asthma, *AA* allergic asthma, *SCIT* subcutaneous immunotherapy, *SLIT* sublingual immunotherapy, *mST* matched symptom treatment group, *ST* symptom treatment

^a^SCIT vs. mST SCIT *p* ≤ 0.05

^b^SCIT vs. mST SCIT *p* ≤ 0.01

^c^ SLIT vs. mST SLIT *p* ≤ 0.05

^d^SLIT vs. mST SLIT *p* ≤ 0.01

In the population with both diseases at baseline the most frequent courses of disease are both diagnoses continuously (31%), omission of both diagnoses (13%), omission of asthma diagnosis followed by continuous rhinitis diagnosis (12%) and remission of rhinitis diagnosis followed by continuous asthma diagnosis (9%). Furthermore patients report omission of asthma diagnosis followed by omission of the remaining rhinitis diagnosis (7%) or reoccurrence of the concomitant asthma diagnosis (7%). Another 6% of the population each show both diagnoses with diagnosis-free periods or omission and reoccurrence of rhinitis. About half of the population with both diseases shows an omission of one diagnosis during the observation period (Table 4).

**Table 4:** Five-year course of disease of insured persons with both diseases (*ARAA*) at baseline by treatment strategy

Description	SCIT	mST SCIT	SLIT	mST SLIT	ST total
*N* total	307 (in %)	614 (in %)	51 (in %)	102 (in %)	20,781 (in %)
*Only ARAA diagnosis* ^*a*^	*33*	*46*	*47*	*47*	*50*
Continuous ARAA diagnosis	21	27	35	31	31
Intermittent ARAA diagnosis^a^	2	6	2	6	6
Omission of ARAA diagnosis	9	12	10	10	13
*Omission of a single diagnosis* ^*a*^	*67*	*54*	*53*	*53*	*50*
*ARAA with omission of AA diagnosis* ^*b,c*^	*41*	*29*	*39*	*24*	*27*
ARAA with omission of AA followed by AR	20	12	22	12	12
ARAA with omission of AA followed by AR and omission	8	8	10	8	7
Intermittent ARAA and AR	11	8	6	4	7
Intermittent ARAA and AR followed by omission	3	1	2	0	1
*ARAA with omission of AR diagnosis* ^*a*^	*20*	*21*	*14*	*23*	*19*
ARAA with omission of AR followed by AA^a^	13	9	8	11	9
ARAA with omission of AR followed by AA and omission^b^	2	4	4	5	4
Intermittent ARAA and AA	5	7	2	7	6
Intermittent ARAA and AA followed by omission	1	1	0	0	1
*ARAA, AR and AA diagnoses* ^*d*^	*6*	*4*	*0*	*7*	*4*
ARAA with intermittent diagnoses of AR and AA	4	3	0	5	3
ARAA with intermittent diagnoses of AR and AA followed by omission	2	1	0	2	1

*AR* allergic rhinitis, *ARAA* allergic rhinitis and allergic asthma, *AA* allergic asthma, *SCIT* subcutaneous immunotherapy, *SLIT* sublingual immunotherapy, *mST* matched symptom treatment group, *ST* symptom treatment

^a^SCIT vs. mST SCIT *p* ≤ 0.05

^b^SCIT vs. mST SCIT *p* ≤ 0.01

^c^SLIT vs. mST SLIT *p* ≤ 0.05

^d^SLIT vs. mST SLIT *p* ≤ 0.01

In the population reporting asthma diagnosis at baseline the most frequent courses of disease are omission of asthma diagnosis (30%), continuous asthma diagnosis (29%) and intermittent asthma diagnosis with diagnosis-free periods (13%). Moreover 7% show occurrence of rhinitis diagnosis followed by its omission. Occurrence of a concomitant rhinitis diagnosis is reported in 28% of the asthma group but only 6% of patients document the additional rhinitis diagnosis continuously.

Due to the small number of patients with isolated asthma in the SCIT, SLIT and mST groups (SCIT *n* = 33; mST *n* = 66; SLIT *n* = 9; mST *n* = 18) a meaningful comparative analysis of the course of disease and medication cannot be conducted.

### Comparison of the course of disease between treatment strategies

The proportion of patients with rhinitis at baseline reporting an additional asthma diagnosis during the observation period does not differ statistically significant between the SCIT group and its matched ST group (Table 3). In the SCIT group patients without additional asthma diagnosis are more likely to show a continuous diagnosis of rhinitis than in the mST group (odds ratio [OR]: 1.37, *p* = 0.003). They also are less likely to report intermittent courses with diagnosis-free years in the SCIT group compared to the mST group (OR: 0.63, *p* < 0.001).

The comparison of the course of disease between the SLIT group and its mST group reveals findings similar to SCIT regarding patients without asthma diagnosis. However, patients under SLIT are significantly more likely to report an additional diagnosis of asthma (OR: 1.94, *p* = 0.031). Due to the small number of patients reporting an additional asthma diagnosis a more detailed assessment of their course of disease is not meaningful (SLIT *n* = 23, ST *n* = 26).

Omission of one diagnosis in patients with both diseases at baseline is reported for a significantly larger proportion of patients in the SCIT group compared to their mST group (OR: 1.74, *p* < 0.001) (Table 4). When one diagnosis is omitted in the SCIT group it is more likely to be the asthma diagnosis (OR: 1.36, *p* = 0.089) and less likely to be the rhinitis diagnosis (OR: 0.65, *p* = 0.024) compared to the mST group. When no omission of a single diagnosis occurs the SCIT group shows a smaller proportion of intermittent courses compared to mST (OR: 0.39, *p* = 0.035), whereas there is no statistically significant difference regarding remissions to diagnosis-free states.

Similar to SCIT, patients with omission of a single diagnosis under SLIT are more likely to lose their asthma diagnosis than those in the mST group (OR: 3.57, *p* = 0.012). Further comparisons of the course of disease between patients receiving SLIT and their mST group are not meaningful due to the small number of patients in these groups (SLIT *n* = 51, ST *n* = 102).

### Comparison of the course of medication between treatment strategies

At baseline the largest proportion of rhinitis patients shows no prescription in the SCIT and SLIT groups (Table 5). In the SCIT group, rhinitis medication is more often documented than asthma medication. Within the SLIT group, rhinitis and asthma medication is prescribed for an equal proportion of patients. In both groups prescriptions for both diseases are rarely documented.

**Table 5:** Medication groups at baseline (2007) for SCIT and SLIT

Medication group	AR		ARAA	
	SCIT^a^ (in %)	SLIT^a^ (in %)	SCIT^a^ (in %)	SLIT^a^ (in %)
No medication	57	66	22	6
Medication for both diseases	7	4	19	21
Medication for asthma	16	15	53	71
Medication for rhinitis	20	15	6	4

*AR* allergic rhinitis, *ARAA* allergic rhinitis and allergic asthma, *AA* allergic asthma, *SCIT* subcutaneous immunotherapy, *SLIT* sublingual immunotherapy

^a^Due to matching by medication group in 2007 the distribution is the same in the matched ST groups

Most of the patients with both diseases hold prescriptions for asthma-related medication followed by medication for both diseases (Table 5). Only 22% of the SCIT group did not have any prescription; within the SLIT group the share was even smaller with 6%. As mentioned above, the isolated asthma group is not analyzed due to the small number of observations.

**Fig. 2:**
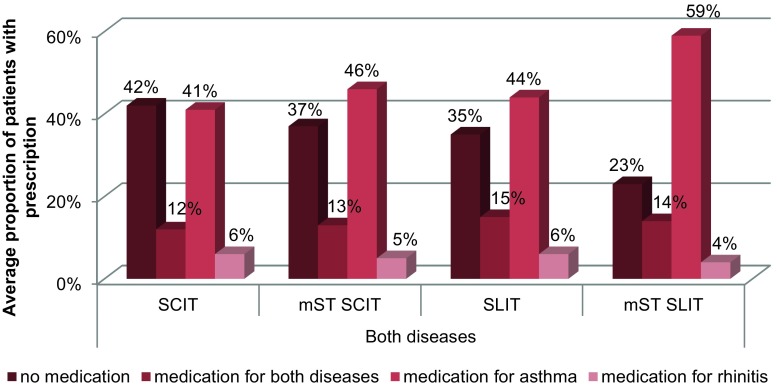
Average proportion of patients with both diseases with disease-related prescriptions during the five year observation period (*SCIT* subcutaneous immunotherapy, *SLIT* sublingual immunotherapy, *mST* matched symptom treatment group)

**Fig. 3:**
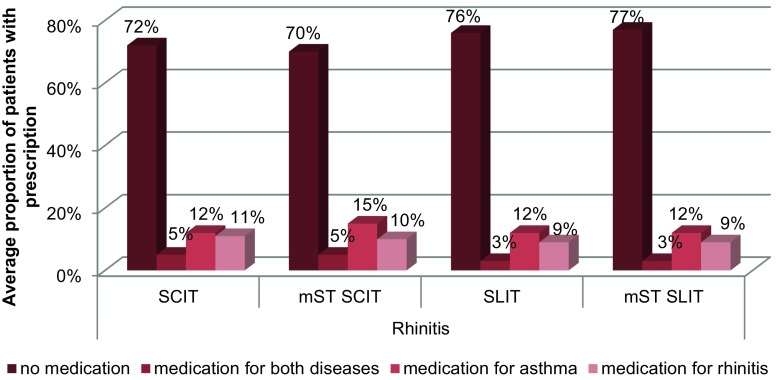
Average proportion of rhinitis patients with disease-related prescriptions during the five year observation period (*SCIT* subcutaneous immunotherapy, *SLIT* sublingual immunotherapy, *mST* matched symptom treatment group)

During the five year observation period the average proportion of patients with both diseases receiving asthma-related prescriptions is lower in the SCIT and SLIT groups than in the mST groups (SCIT 41% vs. 46%, SLIT 44% vs. 59%) (Fig. 2). In patients with isolated rhinitis the average proportion of patients reporting asthma medication is smaller in the SCIT group compared to the mST group (12% vs. 15%) (Fig. 3). In both disease groups there are no relevant differences regarding the average proportion of patients receiving rhinitis medication or medication for both diseases.

In all treatment groups most of the patients with isolated rhinitis hold no disease-related prescription over the observation period (SCIT 37%, mST 39%; SLIT 36%, mST 47%). Among patients with both diseases the largest proportion shows continuous medication during the observation period (SCIT 25%, mST 34%, SLIT 27%, mST 49%). However, compared to mST, rhinitis patients are less likely to receive continuous medication when receiving SCIT (OR: 0.75, *p* = 0.086). Patients with both diseases in the SCIT and SLIT groups are less likely to receive continuous medication than those in the mST groups (SCIT OR: 0.65, *p* = 0.006, SLIT OR 0.39, *p* = 0.011). Furthermore, for patients with both diseases the number of medication-free years differs significantly between the treatment strategies in favor of SCIT and SLIT (*p* = 0.016, *p* = 0.013).

## Discussion

The most frequently documented courses of disease are similar for patients with isolated rhinitis or isolated asthma at baseline: roughly 30% show continuous diagnosis, another 30% report omission of diagnosis and 13–20% have intermittent diagnosis and diagnosis-free periods. About 12% of rhinitis patients and 28% of asthma patients present a second allergic respiratory disease diagnosis during the observation period. In patients with both diseases there is a broader variety of courses of disease: while 30% have continuous diagnosis, 13%, 12% and 9% report omission of both diagnoses, omission of asthma diagnosis and omission of rhinitis diagnosis, respectively. There are also courses of omission and reoccurrence of the omitted diagnosis. In conclusion there are few courses of disease which cover a large share of patients, while the other, smaller share of patients shows a broad variety of courses of disease, sometimes changing disease group every year. This reflects the volatile nature characteristic of the course of allergic diseases.

For rhinitis patients the comparison of the course of disease between SIT and ST overall reveals larger shares of continuous and smaller shares of intermittent diagnosis in the SIT groups. Patients with both diseases in the SIT groups are more likely to report omission of one diagnosis and omission of asthma diagnosis rather than rhinitis diagnosis compared to the ST groups.

Most of the rhinitis patients have no prescribed medication over the observation period, while most of the patients with both diseases report continuous medication. The advantages regarding prescribed medication in the SIT groups compared to ST are the following: a lower average proportion of patients with asthma medication during the observation period, a smaller likelihood of continuous medication and a larger number of medication-free years. These advantages are stronger for patients with both diseases at baseline and for patients receiving SCIT.

Previous findings about the sequence of onset of rhinitis and asthma show that when both diseases occur within one patient this is likely to happen within one year [16, 17]. Supporting these findings, in this study patients with both diseases represent a relevant group (14%) and the share of rhinitis and asthma patients reporting an additional diagnosis over the observation period is small (AR 12%, AA 28%).

The rates of omission of diagnosis in rhinitis and asthma reported in this study (AR 7%, AA 6% per year) are relevantly larger compared to remission rates reported in recently published studies (AR 1.8–4% [13, 18], AA 1.7–2.7% [13, 19, 20] per year). However, there are older studies with remission rates close to or larger than those reported in this study [21–23]. This overestimation of remission might be due to documentation incentives or imprecise coding of the grade of diagnosis-security (“assured”). To our knowledge there has been no study assessing the midterm course of disease in patients with both diseases so far. Therefore, this study adds to closing this gap of knowledge.

There are some general limitations to be considered. First, it should be noted that the presence of a disease is solely based on a diagnosis in one quarter of a year without any further validation. This broader definition of disease allows for consideration of the seasonal occurrence of symptoms as well as for the inclusion of patients treated with nonprescription drugs which is a relevant proportion of rhinitis patients.

The potential bias of this approach is assessed by additional investigation of the course of disease including only patients with disease-related prescriptions at baseline. This analysis did neither reveal relevant changes in the course of disease regarding patients receiving SCIT nor comparing the SCIT group to its mST group. Because of the small number of observations in these subgroups, analyses are not conducted for SLIT.

Second, there might be bias arising from the dependence on diagnosis documentation habits. Due to financial incentives asthma diagnoses are more likely to be documented without being clinically relevant. Therefore the reported omission rates of asthma diagnoses and their difference between the SIT and ST groups should be considered as a conservative estimate. On the other hand, when only the asthma diagnosis is documented while the rhinitis diagnosis is omitted these incentives might lead to an underestimation of the proportion of patients with both diseases. Moreover, there could be over-diagnosing of any disease caused by continuation of diagnosis in the documentation systems without real need of intervention. The treatment-induced more regular physician visits in the immunotherapy groups could result in closer and more accurate documentation leading to a larger proportion of continuous diagnosis and a lower proportion of intermittent diagnosis in these groups.

Third, clinical comparability of the matched ST groups to the immunotherapy groups is limited because no information other than documented diagnoses and prescriptions were available for matching.

Fourth, for symptom treatment of rhinitis several OTC drugs are available for self-medication, which are widely used in these patients. Not including these drugs hinders comprehensive conclusions about the course of medication in allergic rhinitis patients. Furthermore omalizumab is a step five medication for asthma according to international guidelines [10] and SIT would normally not be indicated in patients treated with omalizumab. While no patients in the SLIT and their mST group are treated with omalizumab in the SCIT groups there is a skewed distribution of these patients in disfavor of SCIT (SCIT *n* = 2, 0.2%; mST *n* = 3, 0.1%). However, due to the small proportion of affected patients this is not expected to influence the results of this analysis.

Fifth, despite the large database, the low numbers of patients with isolated asthma and of patients receiving SLIT seriously impair meaningful conclusions regarding the comparison of medication and course of disease between SIT and mST. Several exclusion criteria were inevitable for creating a definite and traceable cohort implying a loss of observations.

To assess the bias of choosing the most frequently reported treatment duration the course of disease is analyzed for patients with a plausible treatment duration not chosen for the main analysis (i. e. four years of SCIT and three years of SLIT). Apart from a slightly disadvantageous course of disease for rhinitis patients receiving four years of SCIT there are no relevant differences between the different treatment durations. Like the main analysis these comparisons are restricted by small numbers of observations in some groups (i. e. asthma and SLIT).

The therapy discontinuation rates of about 40–50% represent another reason for small number of observations in this study. There might be some cases of immunotherapy in which a new prescription is not necessary within one year. Following the definition of discontinuation in this study these cases are wrongly assigned to discontinuation. Therefore, the discontinuation rate might be slightly overestimated.

Excluding patients with immunotherapy prescriptions in the two years prior to the observation period leads to the apparently small percentage of immunotherapy prescriptions reported in this study (about 3%). Analyzing all patients with an allergic respiratory diagnosis in 2007 (*n* = 258,166) reveals that about 7% receive immunotherapy which is still low but comparable to the findings of other studies [24, 25]. The application rate of SIT in asthma patients (2%) is clearly lower than that in patients with both diseases and rhinitis (13%, 7%). The frequency of the SIT application forms and the baseline distribution of disease groups in this study do not differ from those reported elsewhere [24, 25].

Overall, this study provides a detailed overview of the course of treatment and the documented course of diagnoses differentiated by treatment strategy in the German SHI context. Positive effects of immunotherapy on prescribed medication over time are demonstrated, which are expected to be even stronger if nonprescribed medication were included. For patients with both diseases there is an increased likelihood of loss of asthma diagnosis when receiving immunotherapy compared to symptom treatment alone. However, lacking reliable clinical information diagnoses and prescriptions based on health insurance routine data are not sensitive enough to adequately display the benefits potentially achievable through immunotherapy.

## Abbreviations

AA: Allergic asthma
AR: Allergic rhinitis
ARAA: Allergic rhinitis with concomitant allergic asthma
ATC: Anatomical Therapeutic Chemical
ICD: International Classification of Diseases
mST: Matched symptom treatment
OR: Odds ratio
SCIT: Subcutaneous immunotherapy
SHI: Statutory health insurance
SIT: Specific immunotherapy
SLIT: Sublingual immunotherapy
ST: Symptom treatment
GM: German modification
